# Spatially structured bacterial interactions alter algal carbon flow to bacteria

**DOI:** 10.1093/ismejo/wraf096

**Published:** 2025-05-17

**Authors:** Hyungseok Kim, Vanessa L Brisson, John R Casey, Courtney Swink, Kristina A Rolison, Nathan McCall, Amber N Golini, Trent R Northen, Dušan Veličković, Peter K Weber, Cullen R Buie, Xavier Mayali, Rhona K Stuart

**Affiliations:** Department of Mechanical Engineering, Massachusetts Institute of Technology, Cambridge, MA 02139, United States; Physical and Life Sciences Directorate, Lawrence Livermore National Laboratory, Livermore, CA 94550, United States; Physical and Life Sciences Directorate, Lawrence Livermore National Laboratory, Livermore, CA 94550, United States; Physical and Life Sciences Directorate, Lawrence Livermore National Laboratory, Livermore, CA 94550, United States; Physical and Life Sciences Directorate, Lawrence Livermore National Laboratory, Livermore, CA 94550, United States; Physical and Life Sciences Directorate, Lawrence Livermore National Laboratory, Livermore, CA 94550, United States; Environmental Genomics and Systems Biology Division, Lawrence Berkeley National Laboratory, Berkeley, CA 94720, United States; Environmental Genomics and Systems Biology Division, Lawrence Berkeley National Laboratory, Berkeley, CA 94720, United States; Lawrence Berkeley National Laboratory, The DOE Joint Genome Institute, Berkeley, CA 94720, United States; The Environmental Molecular Sciences Laboratory, Pacific Northwest National Laboratory, Richland, WA 99354, United States; Physical and Life Sciences Directorate, Lawrence Livermore National Laboratory, Livermore, CA 94550, United States; Department of Mechanical Engineering, Massachusetts Institute of Technology, Cambridge, MA 02139, United States; Department of Biological Engineering, Massachusetts Institute of Technology, Cambridge, MA 02139, United States; Physical and Life Sciences Directorate, Lawrence Livermore National Laboratory, Livermore, CA 94550, United States; Physical and Life Sciences Directorate, Lawrence Livermore National Laboratory, Livermore, CA 94550, United States

**Keywords:** resource partitioning, photosynthetic carbon fate, metabolomics, stable isotope probing, genome-scale metabolic model

## Abstract

Phytoplankton account for nearly half of global photosynthetic carbon fixation, and the fate of that carbon is regulated in large part by microbial food web processing. We currently lack a mechanistic understanding of how interactions among heterotrophic bacteria impact the fate of photosynthetically fixed carbon. Here, we used a set of bacterial isolates capable of growing on exudates from the diatom *Phaeodactylum tricornutum* to investigate how bacteria-bacteria interactions affect the balance between exudate remineralization and incorporation into biomass. With exometabolomics and genome-scale metabolic modeling, we estimated the degree of resource competition between bacterial pairs. In a sequential spent media experiment, we found that pairwise interactions were more beneficial than predicted based on resource competition alone, and 30% exhibited facilitative interactions. To link this to carbon fate, we used single-cell isotope tracing in a custom cultivation system to compare the impact of different “primary” bacterial strains in close proximity to live *P. tricornutum* on a distal “secondary” strain. We found that a primary strain with a high degree of competition decreased secondary strain carbon drawdown by 51% at the single-cell level, providing a quantitative metric for the “cost” of competition on algal carbon fate. Additionally, a primary strain classified as facilitative based on sequential interactions increased total algal-derived carbon assimilation by 7.6 times, integrated over all members, compared to the competitive primary strain. Our findings suggest that the degree of interaction between bacteria along a spectrum from competitive to facilitative is directly linked to algal carbon drawdown.

## Introduction

Phytoplankton account for approximately half of global photosynthesis [1, 2], so identifying and quantifying the transformations of phytoplankton fixed carbon is fundamental to understanding carbon cycle dynamics. Communities of microbial heterotrophs utilize up to half of the products of photosynthesis [3, 4]. Indeed, phytoplankton exudation is widely acknowledged as a major electron donor for marine microbial respiration as well as a carbon source for microbial growth, and substantial effort has gone into characterizing the exchange of metabolites among phytoplankton and bacteria [5–9]. However, downstream bacteria-bacteria interactions are much less understood and may promote or repress the metabolism of different phytoplankton-derived substrates, resulting in changes to both microbial community composition and respiration. For example, one recent study found that collective microbial respiration is dependent on the interactions among the bacteria within that community [10].

Algal-associated microbial communities exist in an environment known as the phycosphere, that is both spatially and temporally heterogenous [11], and bacteria-bacteria interaction outcomes are dependent on this dynamic microenvironment. For example, motile bacteria that are able to colonize the algal exopolysaccharide matrix may have access to rapid nutrient exchange, relative to their free-living counterparts [11–13]. Algal exudation has been extensively examined for decades [14, 15], and has been shown to vary in quality and magnitude over diel cycles [16] under different nutrient and light stress phenotypes [17] and growth stages [18]. This leads to a broad spectrum of temporal and spatial niche opportunities for different bacterial interactions, and bacterial response to these changes has been extensively shown using ‘omics based approaches (e.g. [19, 20]).

Despite the importance of phycosphere heterogeneity, conceptual frameworks of bacterial interactions do not typically include directionality. Pairwise interactions can be broadly grouped as leading to negative (−), positive (+), or neutral (0) effects for each member of the pair. Negative effects include microbial competition, which can be categorized based on indirect competition for resources (referred to herein as “resource competition”) or direct cell damage (“interference” competition) [21, 22]. Positive interactions can likewise take different forms such as metabolic cross-feeding, production of facilitator exometabolites, or breakdown/uptake of toxic or antimicrobial compounds [23–25]. However, for pairwise interactions, ordered pairs are generally collapsed into a single category, so both 0/+ and +/0 are categorized as “commensal” [26]. We suggest directionality may be important in a spatially or temporally structured environment like the phycosphere, wherein gross growth and resource fluxes of a 0/+ commensal co-culture might differ from a +/0 ordering, for example. To take this into account when considering bacterial competition for algal DOC, we herein refer to “primary” strains that access a portion of algal metabolites first (e.g. proximal to host), leaving an altered pool available to “secondary” strains. By studying metabolic activities of pairwise interactions under different physical contexts, the modes of interaction, their directionality and strengths, and holistic outcomes like resource exchange and growth can be quantified.

Quantifying outcomes of pairwise bacterial interactions in the phycosphere requires a set of metabolically diverse bacteria that have evolved in the dynamic phycosphere spatio-temporal niches, and a cultivation system that allows us to control for directionality. Here, we use a set of 10 bacterial isolates that originate from an outdoor cultivation raceway of the diatom *Phaeodactylum tricornutum* and are common representatives of its microbiome [27, 28]. Seven of the isolates are present in a stable, simplified enrichment community that has been maintained for seven years with *P. tricornutum* in seawater media with no organic carbon added, indicating that these taxa can stably co-exist in a *P. tricornutum*-dependent community [18, 28]. The 10 isolates have distinct metabolic activities, demonstrated by their differential consumption and remineralization of *P. tricornutum* exudates [29], and their distinct effects on *P. tricornutum* physiology [30] when grown in individual co-cultures with the diatom. The isolates also have differential abilities to grow on *P. tricornutum*-derived exometabolites as their sole carbon source [18]. Furthermore, when a *P. tricornutum*-derived exometabolite was added to the enrichment community, several of the taxa which could grow on the exometabolite gained a selective advantage, but others did not behave as predicted, suggesting that other factors aside from resource availability (e.g. bacteria-bacteria interactions) may play a role in governing fitness in this simplified community [18]. To parse competition from other interaction modes, we used a custom porous microplate system. The plate design allows for control over the distance separating bacterial isolates from the alga, and we have previously demonstrated growth of the enrichment community as well as two isolates in the presence of *P. tricornutum* in this system with media containing no added organic carbon [31].

**Figure 1:**
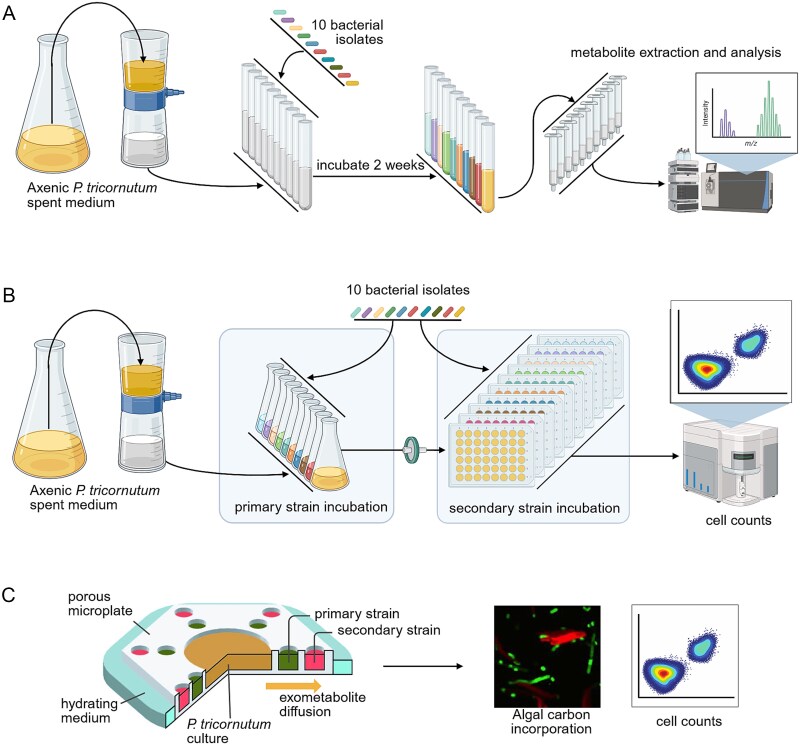
Testing bacterial-bacterial interactions in diatom *P. tricornutum* communities. Experimental procedures to (A) profile differential metabolite consumption and production by each bacterial isolate using untargeted metabolomics, (B) quantify isolate abundance differences through spent medium exchange, and (C) measure isolate activity and bacteria-bacteria interaction in response to algal exometabolites in porous microplates. Created in BioRender. Brisson, V. (2025) https://BioRender.com/dnz243l.

Here, we present three sets of experiments to classify and quantify bacteria-bacteria pairwise interactions in this model diatom-associated community (Fig. 1). The diatom and its exudates are the original source of organic carbon in this system, so we set an assumption of a resource-consumption-based model of community assembly, with resource competition between bacterial taxa as the baseline for all bacteria-bacteria interactions. First, we used metabolomic profiling to predict the degree of potential resource competition between all bacterial pairs. Extracellular metabolites from single bacterial strains grown in *P. tricornutum* spent medium were quantified using solid-phase extraction and liquid chromatography tandem mass spectrometry (LC–MS/MS), a widely used and highly sensitive method for metabolite profiling. This approach has known limitations in detection of small, highly polar compounds and thus our data represent a subset of all possible exometabolites [32], so we applied a complementary prediction approach using genome-based metabolic models. Second, we tested predictions in a sequential spent media experiment, wherein the primary bacterial strain spent media were subsequently fed to each of the other secondary strains in a combinatorial fashion. Finally, to link these interactions to algal carbon fate, we selected two primary strains that had either the most positive or the most negative effect on secondary strains, paired each with the same secondary strain, and quantified algal exudate consumption among the different members, with spatial separation of all three members of the community using the custom porous microplate system [31, 33, 34] and stable isotope probing paired with high-resolution imaging mass spectrometry [35–38].

## Materials and methods

### Strains and culturing conditions

Our study system consisted of one photosynthetic host, the axenic diatom *P. tricornutum* CCMP 2561, and 10 bacterial strains that were previously isolated from an enrichment community from a *P. tricornutum* outdoor mesocosm [27]. Unless stated otherwise, the diatom was cultured at 20°C with a diurnal cycle of 12 h light/12 h dark and a light intensity of 200 μmol m^−2^ s^−1^. The media contained Instant Ocean salts at 20 g l^−1^ added with f/2 inorganic nutrients without silicate (f/2-Si) or artificial seawater medium (ESAW [39]). The culture was transferred to new medium every 2–3 weeks under a biosafety cabinet or laminar flow hood. Bacterial contamination tests were carried out by streaking culture samples on marine broth agar every 2–3 weeks and checking for presence of bacteria using epifluorescence microscopy every 6–12 months. Each bacterial isolate was either maintained in 10% Zobell Marine Broth with Instant Ocean Salts, transferred to new medium every 3–4 weeks, or by co-culturing with *P. tricornutum* in f/2-Si, maintained through monthly transfers for at least 18 months, as described [29].

### Untargeted metabolomics of exometabolite production and consumption

To identify and compare overlap of *P. tricornutum* exometabolites consumed by each of the 10 bacterial isolates, we used an untargeted metabolomics approach to profile metabolites in *P. tricornutum* spent media before and after incubation with each isolate (Fig. 1A).

To remove any residual organic carbon, 500 ml glass Erlenmeyer flasks and 20 ml glass test tubes were baked at 500°C for 2 hours. For algal incubation to obtain spent medium, flasks were filled with 250 ml ESAW and inoculated with one week old *P. tricornutum* culture. Cultures were incubated with shaking (90 rpm, 22°C; 12 h: 12 h, light: dark; 3500 lux illumination) for one week. Cultures were then combined and gently filtered through a 0.22 μm pore-sized polyethersulfone (PES) membrane. Our previous work comparing endo- and exo- metabolites from the same *P. tricornutum* culture showed that this gentle filtration led to significantly distinct pools of metabolites, suggesting minimal cell lysis had occurred [40]. Inorganic phosphate and nitrogen were replenished in the collected spent medium by adding 0.005 g l^−1^ NaH_2_PO_4_.H_2_O, 0.0375 g l^−1^ NaNO_3_ and 0.0236 g l^−1^ NH_4_Cl [18]. From previous work, this spent media contains ~8 ± 2 ppm organic carbon [40]. For bacterial incubation, baked tubes were filled with the 10 ml spent medium and inoculated with 300 μl of bacterial isolate culture at an optical density of 0.15. Five replicates per isolate were inoculated and 12 additional tubes were left uninoculated as a baseline control. Cultures were incubated for two weeks under the same conditions as above. The two-week incubation time was selected to ensure that all strains had sufficient time to grow and consume metabolites was based on the observation of slow growth for many of the isolates on algal spent medium (Supplementary Fig. S1). Although some of the cultures may have been in stationary phase, we did not detect a large number of produced exometabolites, suggesting that lysis was minimal. Cells were removed by filtering the cultures through the 0.22 μm pore-sized filters.

Metabolite samples were extracted from culture filtrates and analyzed using LC–MS/MS after solid phase extraction with Bond Elut PPL columns (Agilent, Santa Clara, CA, USA) as previously described [18]. Metabolite extracts were dried, resuspended in 150 μl methanol containing ^13^C- and ^15^N-labeled matrix control internal standards, filtered through a 0.2 μm pore-sized PES membrane filter and transferred to an autosampler vial. Detailed instrument information and LC–MS/MS conditions and parameters are given in Supplementary Table S6. LC–MS/MS data were analyzed with an untargeted approach. MZMine software [41] was used to identify features based on mass-to-charge ratio (m/z) values and retention times, analyzing positive and negative ionization mode data separately. MZMine analysis parameters are detailed in Supplementary Table S7. Global Natural Products Social Molecular Networking (GNPS) [42] was used to analyze the identified features, conduct molecular networking and putatively identify metabolites.

To compare overlap in consumption between bacterial isolates, we calculated an expected competitive interaction (ECI) between primary and secondary bacterial strains. The LC–MS/MS features (162 features that were identified based on retention time and m/z values and were significantly above background) were further processed to compute ECI (Equation 1) [43–45].


(1) \begin{equation*} {ECI}_{s,p}=-\frac{\sum_m{p}_{m,s}{p}_{m,p}}{\sum_m{p}_{m,s}^2} \end{equation*}

Here *ECI_s,p_* is the (expected) effect on a secondary bacterial strain *s* from a primary bacterial strain *p*, and *p_m,s_* and *p_m,p_* are the proportions of metabolite *m* used by the primary strain *p* and the secondary strain *s*, respectively when grown as an isolate*.* A proportion (e.g. *p_m,s_* and *p_m,p_*) is equal to the ratio in LC–MS/MS signal intensities between isolate-inoculated and the uninoculated spent medium samples, and is determined independently for each isolate [43–45]. Thus, if the primary and secondary strains have the same affinities for all metabolites, i.e. complete competition, then ECI = −1. Alternatively, if there is no overlap between metabolites consumed by primary and secondary strains then ECI = 0. Locations of identified LC–MS/MS features with peak heights, the GNPS results, and custom Python scripts for analyses are provided in Data, Materials, and Software Availability.

### Metabolic Modeling

To compare overlap in metabolite consumption by the bacterial isolates by an independent metric, we used metabolic modeling to predict metabolic resource consumption overlap.

### Metabolic network reconstruction

Bacterial draft genome-scale metabolic models (GEMs) were reconstructed using PheArrMe (https://github.com/jrcasey/PheArrMe), a custom workflow that combines phenotype microarray data with the automated GEM reconstruction pipeline CarveMe [46]. PheArrMe inspects absorption time-series data to determine sole carbon sources and packages those results to guide the CarveMe gap-filling algorithm with known phenotypes. To determine sole carbon sources, 10 bacterial isolates were individually assayed for each of 190 sole carbon sources using 96-well Phenotype MicroArrays (Biolog Inc., Hayward, CA). Growth was monitored by absorbance at 600 nm for 96 hours in a plate reader and analyzed to classify whether a strain consumed a carbon source (Supplementary Note S3). For each isolate, a set of the growth-promoting carbon sources was used to generate a media database along with a base medium composition. The base medium contained macronutrients (NH_4_^+^, PO_4_^3−^, SO_4_^2−^), micronutrients (Co^2+^, Cu^2+^, Fe^2+^, Fe^3+^, Mn^2+^, MoO_4_^2−^, Ni^2+^, Zn^2+^), salts (Ca^2+^, Cl^−^, K^+^, Mg^2+^, Na^+^), and gasses (CO_2_, O_2_). The media database and protein fasta sequences for the enzymes in the metabolic models were passed as arguments to CarveMe.

### Community metabolic modeling

Pairwise competitive interactions were quantified using metabolic resource overlap (*MRO*) [47]. This score reflects the set of minimal nutrient requirements, denoted as *M*, shared between two species, *p*, *s* (Equation 2),


(2) \begin{equation*} {MRO}_{s,p}=\frac{\left|{M}_s\cap{M}_p\right|}{\left|{M}_s\right|} \end{equation*}

The MRO score reflects opportunities for direct competition and does not consider the positive contributions of cross-feeding interactions. It relates most closely to the metabolite production and consumption experiments and is analogous to the ECI score.

### Pairwise bacterial isolate sequential spent media exchange

To test the predictions in metabolic overlap and classify each interaction empirically, we conducted incubation experiments to compare biomass yield of each isolate grown on spent media from another bacterial isolate grown on diatom spent media, in a pairwise fashion (Fig. 1B).

### Incubation and sample collection

800 ml of *P. tricornutum* spent medium was prepared as described above. For the primary strain growth, eleven 125 ml flasks were prepared with 50 ml each of algal spent medium. Each flask was inoculated with 2 ml of a one-week-old (exponential phase) bacterial isolate culture. One flask was left uninoculated as a control. Flasks were incubated under the same conditions as above for two weeks. At the end of the primary strain incubation, cultures were filtered (0.2 μm pore size membrane) to remove cells. For secondary strain growth, eleven 48-well plates (one per secondary strain plus control) were prepared with primary strain spent medium (3 wells per primary strain, 750 μl per well). Each plate well was inoculated with 100 μl of a secondary strain bacterial isolate culture (one isolate per plate) or no isolate (control) and incubated for two weeks. In both cases, the two-week incubation was used to match the metabolomics experiment. At the end of the secondary strain incubation, samples (0.5 ml) were collected for flow cytometry from each well, fixed with glutaraldehyde (final concentration of 0.25%), and stored at −80°C.

### Flow cytometry and analysis

Fixed samples were thawed and diluted in filter sterilized media (10% Zobell Marine Broth with Instant Ocean Salts) to achieve less than 10 000 counts per second. Diluted samples were aliquoted (250 μl) into a 96 well plate and stained with 2.5 μl of 100X SYBR Gold for 10–15 minutes in the dark. Cells were counted on an Attune benchtop flow cytometer as described previously [29]. Blanks consisting of ultrapure water were run between each set of biological triplicates.

Using the bacterial cell counts, a sequential interaction (SI) effect representing the influence of a primary strain on a secondary strain was calculated (Equation 3) [43].


(3) \begin{equation*} {SI}_{s,p}=-\frac{G_{s, PtSM}-{G}_{s,p}}{G_{s, PtSM}} \end{equation*}

Here *SI_s,p_* is the effect on a secondary strain *s* from a primary strain *p*, *G_s,PtSM_* is the growth (in cell counts) of secondary strain *s* on *P. tricornutum* spent medium, and *G_s,p_* is the growth of secondary strain *s* on spent medium from primary strain *p.* The difference between *G_s,PtSM_* and *G_s,p_* was normalized by the secondary strain growth so that SI strength scaled to −1 for a complete competition or 0 for no competitive impact. Unlike ECI, SI can also be positive because it can capture facilitative as well as competitive interactions.

### Porous microplate co-culture

To address the hypothesis that different bacterial interactions lead to distinct carbon flow, we used porous microplates to co-incubate either a representative competitive primary strain (with relatively low ECI and SI values, and high MRO) or a representative facilitative primary strain (with positive SI values) with the same secondary strain, and compared algal carbon incorporation in the secondary strain (Fig. 1C).

### Incubation and sample collection

To quantify the algal carbon transferred to primary and secondary bacteria consumers, we used a stable isotope labeling approach. As previously described [31], axenic *P. tricornutum* was acclimated to the copolymer by inoculating stationary phase cells into a microplate (see Supplementary Note S1 for device preparation). The cells were incubated for a week and were diluted four times using f/2-Si containing 2 mM ^13^C sodium bicarbonate (Cambridge Isotope, 98 atom%) and 10 nM ^15^N leucine (Cambridge Isotope, 98 atom%). Diluted cells were inoculated into the center well of a microplate at a starting concentration of 8.4 × 10^6^ cells ml^−1^. For bacteria, each colony of *Alcanivorax* sp. EA2, *Devosia* sp. B7WZ and *Marinobacter* sp. 3–2 was inoculated into marine broth and grown overnight at 30°C, 250 rpm. Overnight cultures were washed twice with f/2-Si, left overnight at room temperature and diluted to OD600 ~ 0.01 with isotope-containing f/2-Si. Diluted cells were inoculated into surrounding microplate wells. Porous microplates and the cells were immersed in f/2-Si with the isotope. On Days 5 and 14 post incubation, 35 μl bacteria and 300 μl *P. tricornutum* were collected from the microplate. Bacterial cells were subsampled and streaked on marine broth agar to confirm presence and to test for cross-contamination. Remaining samples were fixed using formaldehyde with a final concentration of 2% v/v. Fixed cells were left at room temperature for 1 day and subsequently stored at 4°C up to 8 weeks.

### Flow cytometry

Forty microliters of fixed bacteria were added with 0.1 μl SYBR Green I nucleic acid stain and were allowed to sit for 0.5–1 h at room temperature without light exposure. To each well containing the stained cells, 2 μl flow cytometry counting beads and 158 μl 0.1 M TAPS buffer (pH 7.76) were added, bringing to a total volume of 200 μl. Flow cytometry was conducted on a BD FACS Canto II HTS and a BD FACS Diva software (Supplementary Table S9). Events were collected and clustered based on FITC-A and Alexa Flour 680-A gates. The count numbers were exported as csv files and analyzed using Microsoft Excel or R.

### NanoSIMS imaging analysis

Twenty microliters were subsampled from each fixed sample with cells collected on Day 14 post incubation. For each microplate and treatment, triplicates were pooled to bring to 60 μl in total and filtered on a small area of a 0.2 μm pore size polycarbonate membrane (Whatman Nuclepore, Cytiva, Marlborough, MA). Filters were rinsed, dried, the filtered areas cut and adhered to conductive carbon tape (Ted Pella, Redding, CA), gold coated, and analyzed by NanoSIMS as previously described [27, 29] (Supplementary Note S2). Secondary ion images were collected for masses ^12^C^12^C^−^, ^12^C^13^C^−^, ^12^C^14^N^−^, ^12^C^15^N^−^, and ^32^S^−^ on individual electron multipliers, as well as secondary electrons (SE). All nanoSIMS images were processed using L’Image software to correct for dead time and image shift across cycles, create ^13^C/^12^C and ^15^N/^14^N ratio images, and draw regions of interest (ROIs) to measure bacterial ^13^C and ^15^N incorporation. We calculated isotope ratios ([^12^C^13^C^−^ / ^12^C^12^C^−^] / 2 = ^13^C / ^12^C) of each bacterial cell [35]. Based on the algal ^13^C labeling, the percent of bacterial carbon derived from the alga was calculated (net carbon incorporation, C_net_) [29, 36, 48]. The isotope composition of algal exudate could differ from the measured ^13^C enrichment of the algal cells, so herein C_net_ is considered as a *comparative estimate only* of bacterial incorporation. Algal cells were similarly abundant and ^13^C enriched across treatments (Supplementary Fig. S9), which should lead to similar levels of exudate isotope enrichment. A total incorporation of algal C mass by bacterial isolates was defined and calculated (Supplementary Note S2), by combining the single cell C_net_, abundances and cell size across the microplate culture wells.

### Matrix assisted laser desorption/ionization imaging of algal and bacterial metabolites

To compare primary strain activity that may support cross-feeding, we used matrix assisted laser desorption/ionization (MALDI) to identify bacterial-produced metabolites for the representative strains.

### Sample preparation

Axenic *P. tricornutum*, three bacteria only (*Devosia*, *Alcanivorax*, and *Marinobacter*) and three co-cultures (*P. tricornutum* with *Devosia*, with *Alcanivorax*, and with *Marinobacter)* were incubated in liquid f/2 media for one week. Ten microliters of each culture were spotted onto f/2 agar and allowed to dry for 10 minutes. The plates were wrapped in parafilm and incubated under a 14 h:10 h light/dark cycle at 22°C for 10 days. Details of MALDI mass spectrometry imaging (MSI) and analysis are in Supplementary Note S4.

## Results

### Metabolite consumption patterns and metabolic models predict degree of resource competition

**Figure 2:**
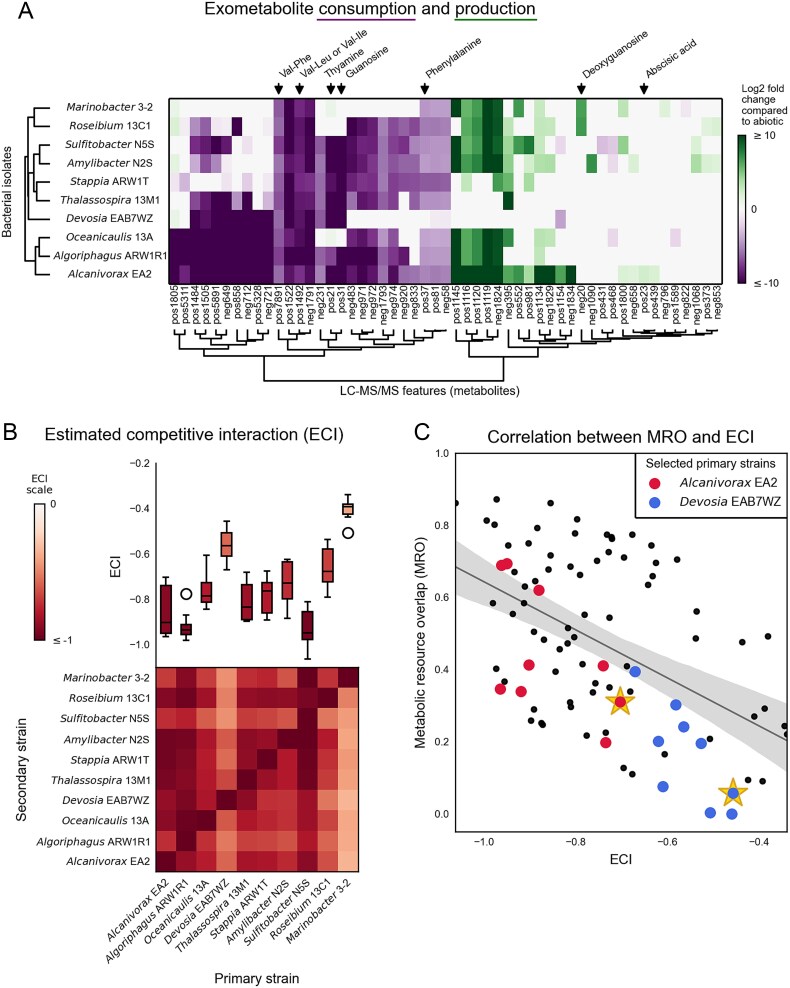
Predicted bacterial resource competition for algal exometabolites. (A) Metabolite consumption and production by bacterial isolates. Columns represent each metabolite, with putative identifications (based on MS/MS spectral matches to GNPS) indicated at the top. Rows represent each bacterial isolate inoculated into *P. tricornutum*-spent medium. Shading represents statistically significant (*P* ≤ .05 for Student’s *t*-test with Bonferroni correction) consumption or production of each metabolite compared to the levels in uninoculated *P. tricornutum* spent medium according to the scale bar. Shading lightness/darkness indicates the extent of consumption or production (log_2_ fold change compared to uninoculated *P. tricornutum* spent medium), with darker shading indicating greater changes. No Shading indicates no significant difference. Left dendrogram shows hierarchical clustering of bacterial isolates based on metabolite feature consumption and production profiles. Bottom dendrogram shows hierarchical clustering of LC–MS/MS features (B) ECI based on metabolite consumption overlap. Heatmap shows the ECI for each primary strain (columns) on each secondary strain (rows). Darker shading indicates stronger competitive interactions. Top boxplot summarizes ECI for each primary strain (column). In the boxplot, the middle line indicates the median, the box shows the inner-quartile range, the whiskers indicate the farthest data points within 1.5 times the inner-quartile range, and the circles indicate any data points beyond the whiskers. Boxplot shading indicates median ECI values according to the scalebar. (C) Correlation between MRO and ECI. Each point represents one primary-secondary pair. Primary strains studied in the porous microplate experiment are indicated with larger, differently shaded points for *Alcanivorax* and *Devosia*. Pairings of those primary strains with the secondary strain studied in the microplate experiment, *Marinobacter*, are highlighted with stars. The diagonal line shows the linear correlation (Pearson’s *R*^2^ = 0.25, *P* = 6 × 10^−7^), and the shaded area indicates the 95% confidence interval. Strength of resource overlap is predicted by the metabolite patterns and the metabolic models.

We used an extracellular metabolomics approach to predict metabolite consumption overlap (i.e. resource competition) between the 10 bacterial strains. *P. tricornutum* was grown on a fully defined seawater media (ESAW) without any organic carbon, the spent media was filtered to remove cells, each of the bacterial strains was grown separately on the spent media, and the resulting media were analyzed with untargeted metabolomics and compared to uninoculated spent media (Fig. 1). Bacteria consumed a subset of algal exometabolites (Fig. 2). Our analysis detected a total of 162 LC–MS/MS features (hereafter referred to as metabolites), based on retention time and m/z values, that were significantly (Bonferroni adjusted *P* value <.05 from Student’s t-test) above background compared to extraction blanks for at least one sample group (Supplementary Table S1, Supplementary Fig. S2). Of these 162 metabolites, 54 (33%) had statistically significant changes in concentration (either increased or decreased in relative signal intensity) for at least one bacterial isolate compared to uninoculated *P. tricornutum* spent medium (Fig. 2A). Herein we define bacterial consumption of a metabolite by the significant decrease in signal intensity, which could be explained by different bacterial-mediated mechanisms such as incorporation into biomass, respiration, or metabolite modification. We detected only 23 metabolites that were produced (here defined as significantly increased compared to algal spent medium) across the bacterial isolates (Fig. 2A, Supplementary Fig. 1), suggesting that bacterial cell lysis was not a major contributor to the overall metabolite pool at the end of the incubation. Hierarchical clustering grouped the bacterial isolates based on their patterns of production and consumption (Fig. 2A, left dendrogram). A Mantel test [49] was conducted to assess whether the patterns of metabolite consumption correlated with bacterial phylogeny, and no phylogenetic correlation was detected (*r* = 0.11, *P* = .29). Of the 54 changing metabolites, seven (13%) could be putatively identified based on their MS/MS spectra (Table 1, with identification details in Supplementary Table S2). Only four metabolites were depleted by at least half (log_2_ fold change ≤ −1) by all bacterial isolates, two of which were putatively identified as dipeptides.

**Table 1:** Putative identifications (based on MS/MS spectral matches to GNPS) of consumed or produced metabolites.

Metabolite Feature ID	Putative Identification	Consuming strains	Producing strains
Positive-1492	Val-Leu or Val-Ile	All isolates	
Positive-7891	Val-Phe	All isolates	
Positive-21	Thymine	All isolates except *Marinobacter* 3–2, *Labrenzia* 13C1 and *Oceanicaulis* 13A	
Positive-31	Guanosine	All isolates except *Marinobacter* 3–2, *Labrenzia* 13C1 and *Oceanicaulis* 13A	*Marinobacter* 3–2 and *Oceanicaulis* 13A
Positive-37	Phenylalanine	All isolates except *Devosia* EAB7WZ	
Negative-20	Deoxyguanosine		*Marinobacter* 3–2 and *Labrenzia* 13C1
Positive-23	Abscisic acid		*Alcanivorax* EA2

We characterized the overlap between metabolite consumption patterns by calculating an ECI coefficient between primary and secondary strains [43–45], where the primary strain is defined as the strain that would hypothetically have access to the algal metabolites before the secondary strain. An ECI value of 0 indicates no competition from the primary strain and − 1 indicates complete competition, where both primary and secondary strains have the same ability to degrade all metabolites (see Eq. 1), as is the case when the primary and secondary strains are the same (intraspecific competition). We found a wide range of predicted resource competition (Fig. 2B). For instance, *Marinobacter* sp. 3–2 (hereafter referred to as “*Marinobacter”*) and *Devosia* sp. EAB7WZ (hereafter referred to as “*Devosia*”) as the primary strains had the weakest (least negative) average ECI coefficients, suggesting they are less likely to compete with the other strains. By contrast, three other strains (*Alcanivorax* sp. EA2, *Algoriphagus* sp. ARW1R1, and *Sulfitobacter* sp. N5S) showed stronger average competitive interaction coefficients. As secondary strains, most isolates had a highly variable ECI, depending on the primary strain, as expected.

As a complementary predictive metric, these strains have genomes available, thus we independently calculated the potential MRO. MRO is an estimate of resource uptake potential between two metabolic networks (a value of 1 indicates that the primary strain can take up all resources that the secondary strain can; a value of 0 indicates it can take up none; Eq. 2). A comparison of the MRO and ECI scores provides a metric for the comprehensiveness of our mechanistic understanding of purely competitive interactions. There was weak but statistically significant agreement with the ECI-based estimate (Fig. 2C, Supplementary Fig. S3). The MRO and ECI were significantly negatively correlated (Pearson’s *R*^2^ = 0.25, *P* = 6 × 10^−7^), indicating that a modest portion of the observed competitive interactions could be predicted based on known metabolic potential alone.

### Sequential bacterial interactions were more positive than predicted from resource competition

**Figure 3:**
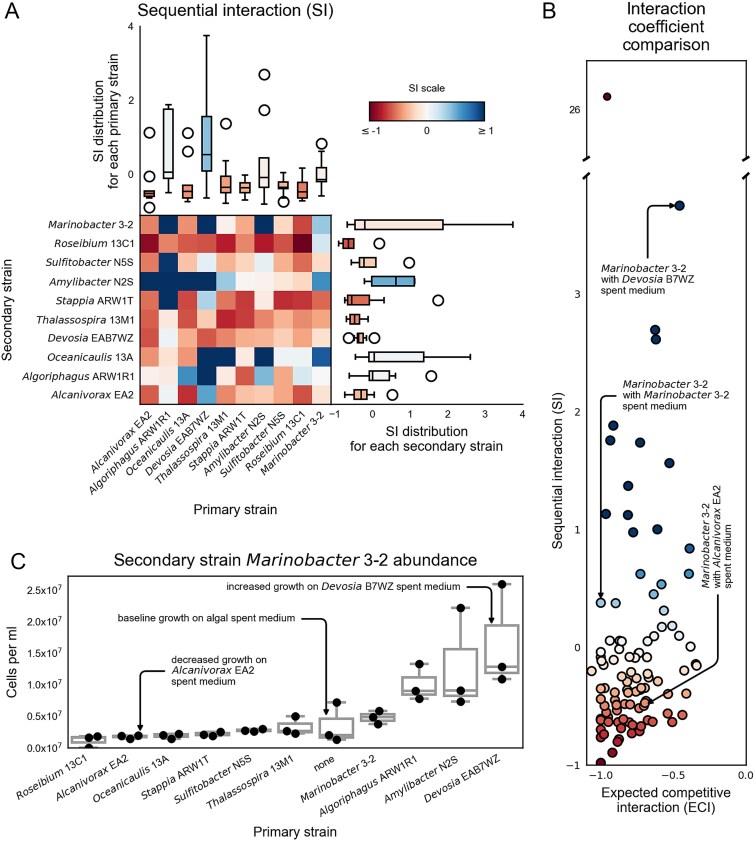
Sequential interaction (SI) response and comparison with ECI predictions. (A) SI based on growth from sequential spent medium exchange. Heatmap shows the SI for each primary strain (columns) on each secondary strain (rows). Darker shading indicates stronger interactions according to the scale bar. Top boxplot summarizes SI for each primary strain (column). Right boxplot summarizes SI for each secondary strain (row). In the boxplots, the middle line indicates the median, the box shows the inner-quartile range, the whiskers indicate the farthest data points within 1.5 times the inner-quartile range, and the circles indicate any data points beyond the whiskers. Boxplot shading indicates median SI values. (B) Comparison of measured sequential interaction (SI) strengths to ECI strengths. Each point represents the SI and ECI for one primary-secondary bacterial pair. Point shading indicates the strength of the SI, according to the same scale bar as in part (A). Annotations indicate bacterial isolate pairs selected for porous microplate experiments. Sequential spent medium exchange suggests the presence of outliers and other bacterial-bacterial interactions. (C) Final cell counts for *Marinobacter* 3–2 as the secondary strain on spent media from the different primary strains and on algal spent medium (no primary strain processing).

After obtaining two estimates of resource competition among the algal-associated bacteria (one experimental, the other theoretical), we conducted a sequential experiment to test these predictions and identify the prevalence of bacteria-bacteria interactions other than resource competition (Fig. 1B). This experiment tested how growth of a primary strain on *P. tricornutum* spent medium affected the growth of a secondary strain in the second stage of the experiment. We tested all 10 strains as primary and secondary consumers, including experiments with the same strain as primary and secondary, testing intraspecific interactions. We calculated a sequential interaction coefficient (SI) from the cell counts of the second strain where −1 reflected no growth, 0 reflected the same growth, and 1 reflected twice the growth as was observed without the primary strain (Eq. 3; Fig. 3A, Supplementary Table S3) [43]. Unlike ECI which only captures competition and is thus always negative, SI reflects both competition and facilitation by the primary strain and can thus have positive values.

The sequential interaction results indicated both competitive and facilitative interactions were present. Of the 100 primary-secondary strain pairs tested, 70 had a negative SI coefficient, suggestive of competition-dominated interaction. However, the results also indicated the importance of other factors, such as facilitation, contributing to less negative interactions than predicted from resource competition alone, and to positive SI coefficients for the other 30 primary-secondary strain pairs, reflecting growth promotion of the secondary strain by the primary strain (Fig. 3B). *Devosia* in particular exhibited a majority of facilitative interactions as the primary strain, improving the growth of 6 of the 9 tested secondary strains, and a positive average SI value (Fig. 3A, C, Supplementary Fig. S10). A comparison of ECI and SI for all pairwise combinations of primary and secondary strains showed that 97% of SIs were less negative than the corresponding ECIs, further suggesting that while competition may dominate the net response, facilitative activity may occur for many strain pairs.

Overall, we found that SI was weakly correlated with ECI across all 100 primary-secondary strain pairs (Spearman’s *r* = 0.27, *P* = .0068) (Fig. 3B). When aggregated by primary strain, we did not find consistent patterns in the relationship between SI and ECI across all primary strains (Spearman’s *r* = 0.22, *P* = .53) (Fig. 2B and Fig. 3A, top bar charts). However, some primary strains showed consistent patterns between ECI and SI. For example, *Alcanivorax* sp. EA2 (hereafter referred to as “*Alcanivorax*”) had low (more negative) SI and ECI coefficients (Fig. 2B, and Fig. 3A, top bar charts, Supplementary Fig. S10).

### Custom porous microplate experiments to quantify bacterial interactions

**Figure 4:**
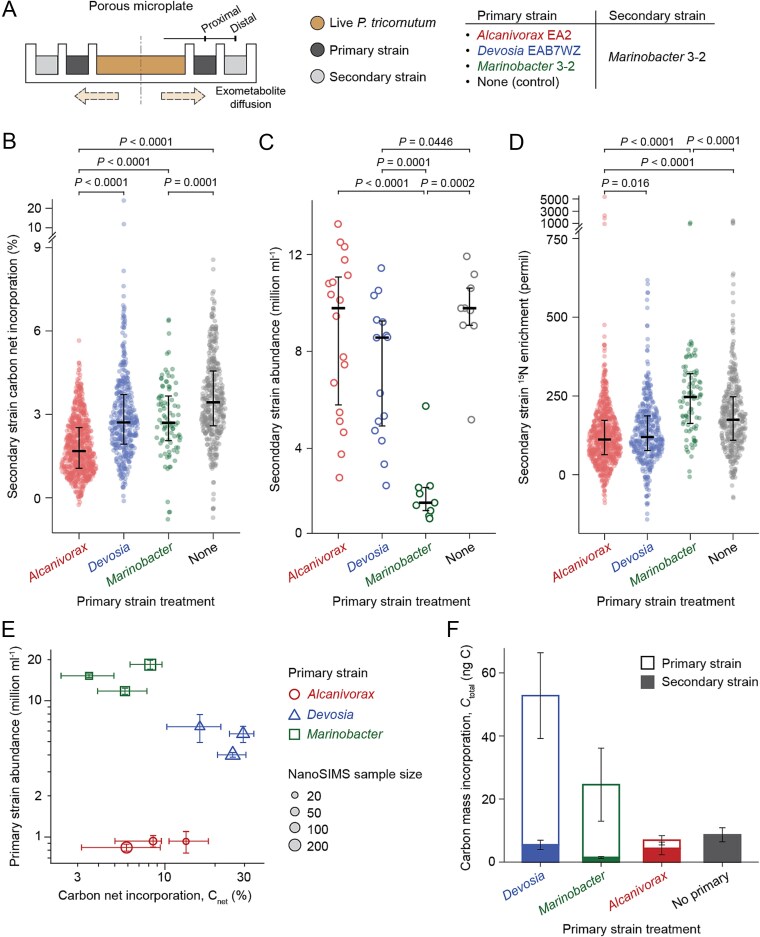
Porous microplate to test interspecies interactions. (A) Side view schematic of porous microplate for co-culturing *P. Tricornutum* and primary-secondary pairs. The microplate was designed such that algal C flux to bacteria is the closest to the algal-bacterial co-culture (Supplementary Note S1). (B) Net carbon incorporation (C_net_) by secondary strain *Marinobacter* on Day 14. Each point represents a single cell NanoSIMS measurement. (C) Abundance of the secondary strain *Marinobacter* on Day 14, co-cultured with primary strains and *P. Tricornutum* in the porous microplate. Each point represents the number of cells in a microplate well. (D) Enrichment of ^15^N by secondary strain *Marinobacter* on Day 14. Each point represents a single cell NanoSIMS measurement. Black lines indicate median (thick) and interquartile range (error bar). (E) Relationship between bacterial abundance and single cell C_net_ for primary strains after 14 days. Each symbol represents measurement from a single microplate. Vertical and horizontal error bars respectively denote standard deviation of cell number and the first/third quartile of C_net_. (F) Estimate of total incorporation of the carbon mass (*C*_total_) by bacterial pair (see Supplementary Note S2 for derivation). Error bars denote standard deviation of three microplate replicates. The microplate co-culture confirms the presence of bacterial interactions with live *P. Tricornutum*.

The ECI, MRO, and SI scores identified competitive and facilitative interactions between pairs of bacteria with algal spent media as their sole carbon source. To test the hypothesis that these competitive and facilitative interactions led to distinct carbon flow into microbial populations, we co-incubated primary-secondary pairs in the presence of live algae and quantified algal carbon fate (Fig. 1C). We chose the most positive primary strain, *Devosia*, and the most competitive primary strain, *Alcanivorax*, each incubated with the same secondary strain, *Marinobacter* (Fig. 3A). We further classified *Devosia* as facilitative because the majority of its SI values were positive (6 of 9, excluding self-interaction), demonstrating its growth-enhancing effect on these bacterial strains. *Alcanivorax* was chosen because it had the lowest ECI and MRO average scores, its sequential interactions best matched ECI and MRO predictions, and it consumed the most diverse set of metabolites, suggesting that resource competition may be the dominant effect in its interactions with other bacteria. *Marinobacter* was chosen as the model secondary strain as it had the broadest range of secondary strain SI values (Fig. 3A, Fig. 3C), indicating it is sensitive to primary strain activity. We also included a positive control with *Marinobacter* as both the primary and secondary strain (intraspecific resource competition), and a negative control with no primary strain in the proximal wells (Fig. 4A).

These experiments were conducted in custom porous microplates designed to mimic a diffusion gradient around an algal cell (Supplementary Fig. S4, Supplementary Note S1). Through spatial structuring, the design allowed primary strains first access to continuously released exudates from live algae, while excluding physical interactions (i.e. attachment) between all members. *P. tricornutum* was incubated in the central well of the porous microplates, the primary strain in wells proximal to the center, and the secondary strain in distal wells further from the center (Fig. 1C). A previous study using this porous microplate indicated that at least 14 days were required for the bacterial strains to reach exponential growth [31], which informed our selection of the sample collection point [50]. To track photosynthetically fixed carbon and examine bacterial growth, ^13^C-labeled bicarbonate and ^15^N-labeled leucine [51] were added to the incubations. Primary strains in the proximal wells incorporated a higher amount of algal carbon than secondary strains in the distal wells (Supplementary Fig. S5), supporting our microplate design configuration.

### Secondary strain incorporates more algal carbon with a facilitative primary strain than a competitive strain

From single cell ^13^C enrichment values quantified by NanoSIMS, we calculated the percent of bacterial carbon derived from algal photosynthesis (C_net_ [29, 36, 48]). Median C_net_ for secondary strain *Marinobacter* with facilitative primary strain *Devosia* was 1.6 fold higher (2.71%) than when resource-competitive *Alcanivorax* was the primary strain (1.69%, *P* < .0001, Wilcoxon test) (Fig. 4B). This supports our predictions and provides a quantitative metric for algal carbon acquisition gains or losses when a bacterium is in the presence of a positive or negative interactor. The negative control, with no primary strain in the proximal well, exhibited the highest secondary strain C_net_ (3.44%), showing that the presence of any primary strain in the proximal ring lowered the carbon incorporation of secondary strain *Marinobacter* cells (*P* < .001, Kruskal-Wallis test). This suggests that even our facilitative primary strain removed or altered some carbon substrates that *Marinobacter* would otherwise consume. We did not observe a significant effect on the distal *Marinobacter* abundance in the presence of proximal *Devosia* or *Alcanivorax* relative to the negative control (*P* = 0.045, 0.781, Wilcoxon test, Fig. 4C). This points to the sensitivity of carbon incorporation compared to abundance measurements. It also suggests that the degree of *Devosia*’s facilitative activity, which was significant in the sequential media experiment but not significant based on abundance in the microplate experiment, depends on substrates or conditions that were different between the sequential media and microplate experiments.

The positive control with *Marinobacter* as both primary and secondary strains (intraspecific resource competition) did not show reduced carbon incorporation per cell relative to other primary strain treatments. However, examining bacterial abundances in the wells at exponential phase (e.g. Day 14 as previously found [31], and supported by increases in abundance between Day 9 and Day 14 Supplementary Fig. S6), we found that with intraspecific competition, secondary strain *Marinobacter* exhibited significantly lower abundances relative to the other treatments by 4.9–5.6 fold (*P* < .001, Wilcoxon test, Fig. 4C, Supplementary Fig. S6). This indicates that intraspecific competition led to lower biomass yield but similar per cell algal carbon incorporation, and suggests a different response to intraspecific and interspecific resource competition for *Marinobacter* in which intraspecific competition led to significantly reduced biomass yields and interspecific competition allowed an ability to compensate with alternative carbon substrates.

In addition to quantifying the transfer of algal carbon into bacteria cells, we also examined two other independent measures of growth and physiology to compare *Marinobacter* incubated with different primary strains: uptake of ^15^N-labeled leucine and cell size. Distal *Marinobacter* cells were more ^15^N enriched in the presence of *Devosia* compared to *Alcanivorax* (*P* = .016), and significantly less enriched than the negative control with no primary strain (*P* < .001, Fig. 4D), similar to the C_net_ results. However, the intraspecific competition *Marinobacter* treatment deviated from the C_net_ results, in that those cells had the highest ^15^N enrichment of all distal wells, and also higher enrichment than the proximal well *Marinobacter* cells (Supplementary Fig. S7). Cell size also differed for this positive control, where proximal *Marinobacter* cells were larger than those in the distal well (*P* < .001, Wilcoxon test), but the secondary strain cell size was not statistically different from the negative control with no primary strain (*P* = .84, Supplementary Fig. S8). Together, the low cell abundances, smaller cell size, similar C_net_ and higher ^15^N enrichment of the intraspecifically interacting distal wells, may indicate that the cells were entering a starvation state [52]).

Primary strain C_net_ and abundances varied significantly across the treatments (Fig. 4E), likely a result of strain-specific carbon use efficiency. *Alcanivorax* had lower cell abundances and carbon incorporation than *Devosia* (median C_net_ of 6.7% vs 26.0%), suggesting that *Alcanivorax* was an efficient competitor, because it was still able to negatively affect the carbon incorporation of the *Marinobacter* secondary strain (Fig. 4B) despite low activity. To compare total algal carbon transfer to bacteria (both the secondary and primary strains), we combined cell counts, cell size, and single cell carbon assimilation measurements to calculate total carbon incorporation in the entire microplate (both distal and proximal wells) over the incubation period, denoted as *C*_total_ (Supplementary Note S2). Among the three tested pairs of isolates, the combination of *Devosia* and *Marinobacter* had a 7.6 fold higher total incorporation of algal carbon relative to *Alcanivorax*-*Marinobacter* (Fig. 4F).

### Metabolite production by the model primary strains

The sequential spent media interaction results showed that *Marinobacter* growth was enhanced by *Devosia* resource processing (Fig. 3C) but the LC/MS metabolomics analysis did not detect any metabolites produced by *Devosia* (Fig. 2A). Also, the microplate results did not confirm a significant net facilitative effect, suggesting that either *Devosia* produced undetected metabolites or *Devosia* facilitation occurred through a non-cross-feeding based mechanism, such as degradation of an inhibitor that may have had differing concentrations between the sequential media and microplate experiments. To address the first hypothesis, we conducted a matrix assisted laser desorption and ionization (MALDI) mass spectrometry metabolite imaging experiment to detect compounds that might have been lost during the solid phase extraction prior to LC–MS/MS analysis. MALDI imaging allowed us to compare total metabolite production (endo and exometabolites) by *Alcanivorax*, *Devosia*, and *Marinobacter* incubated on solid medium with no added organic carbon source in isolation and adjacent to *P. tricornutum*. Overall, 246 metabolites were detected from at least one bacterial isolate (Supplementary Table S5). When incubated on solid medium, *Alcanivorax* and *Marinobacter* produced more metabolites (42 and 41 detected metabolites respectively) than *Devosia* (13 detected metabolites), similar to the LC–MS/MS exometabolites analysis. Of these, 30 metabolites were unique to *Alcanivorax* and 20 were unique to *Marinobacter*, but none were unique to *Devosia*. LC–MS/MS metabolite profiling and MALDI data were complementary, capturing distinct sets of metabolites, and moreover the experiments represented growth on liquid and solid media, respectively, so these results suggest that the mechanism of facilitation observed in the sequential spent media experiment was likely not cross-feeding.

## Discussion

We designed a series of experiments to characterize how different interactions between heterotrophic bacterial isolates found to co-exist in *P. tricornutum*-associated communities [18] influence algal carbon fate. Through profiling of all pairwise interactions between isolates, we found that facilitative interactions were prevalent, with 30% of the pairwise interactions leading to growth promotion by a primary strain. Informed by these profiling results and predictions of resource overlap, we chose three primary strains to compare carbon incorporation into a secondary strain. This allowed us to quantify how much carbon the secondary strain lost access to due to consumption or modification by the primary strain, providing a metric for the amount of lost resources due to competition, which we refer to as a cost. Our three primary strains fell along a spectrum of interaction representing (1) a facilitative interaction with a positive SI value, (2) a competitive interaction with high metabolic overlap based on ECI and MRO values, and (3) an intraspecific interaction. The competitive interaction (*Alcanivorax*) led to a 51% reduction in carbon drawdown per cell for the secondary strain, compared to no interaction, indicating a significant cost of competition. The facilitative interaction treatment (*Devosia*) also resulted in a cost in terms of carbon drawdown, with 21% less carbon drawdown per cell than no interaction. Although this cost was significantly lower than that observed in the competitive treatment, it suggests that resource competition was occurring in this interaction as well, and that the degree of facilitation may be dependent on context, such as the concentration of specific exometabolites, or the presence of the diatom. While bacterial communities consuming phytoplankton-derived dissolved organic carbon (DOC) appear to have a high degree of resource partitioning [53], and host resources alone have been shown to be predictive of algal-associated microbial community assembly in a synthetic system [54], applying the concept of resource partitioning alone often does not accurately predict the outcome of resource consumption. For example, the quantity of phytoplankton-derived DOC has been shown to be an important factor in determining the outcome of bacterial consumption [55]. Furthermore, incorporation of bacteria-bacteria interactions into models can improve disparities from predictions based solely on consumer-resource models [56], suggesting the importance of these interactions for carbon fate predictions. Research on plant phyllosphere communities indicate that bacterial resource competition can be predicted by metabolic overlap, but that degree of resource competition is reduced by spatial heterogeneity of the leaf environment compared to the in vitro conditions [57]. Our experiments similarly demonstrate that metabolic overlap can predict resource competition for algal organic carbon, and since our microplates captured some of the dynamics of the phycosphere environment by exposing the bacteria to exudates from a live diatom culture, along with controlling directionality, the costs measured represent a more relevant estimate of degree of resource competition. Our results exemplify how, when directionality is controlled, we can quantify the impact of different interactions on the flow of carbon in the context of dynamic exudation from live algae. Although further testing will be required including cultivation with physical interaction, these results also suggest that this carbon cost could be predicted based on bacterial interaction in a spatiotemporally relevant system.

Carbon assimilation was distinct between the two model primary strains, highlighting the importance of activity-based measurements to examine interactions. We expected similar carbon assimilation based on our previous work tracing ^13^C-labeled *P. tricornutum* solid-phase extracted exudate into co-cultures, where daily percentage of biomass C assimilated from exudates was similar between the two strains (C_net_ of 1.7% and 2.3% for *Alcanivorax* and *Devosia*, respectively [29]). When expanded to examine carbon flow in the whole microplate, *Devosia* had 2.7 fold higher carbon assimilation and 6.0 fold higher cell abundances than *Alcanivorax*, leading to an amplified effect on total carbon incorporation. Although metabolic profiling did not predict this difference, respiration rates can differ by orders of magnitude for different marine taxa, and abundance can be decoupled from respiration rate [58], so this could explain differences in metabolic efficiencies between the primary strains. For example, *Alcanivorax* can consume a diverse array of substrates, but this may lead to low bacterial growth efficiency (high respiration), consistent with the metabolic and proteomic respiratory burdens of copiotrophs [59] as well as our measurement of its low leucine uptake (Supplementary Fig. S7). Although metabolic modeling can predict the spectrum of biomass yields across exogenous carbon sources, without constraints on uptake, estimates of the total carbon use efficiency are unreliable. A promising strategy to estimate uptake couples quantitative exometabolomics to metabolic models that resolve transporter kinetics (e.g. Boundary Flux Analysis [60]). Another explanation could be the limitations of detection by our exometabolomics approach which generally does not capture small polar compounds including glucose, or larger molecular weight molecules such as polysaccharides [32]. Regardless, the three primary strains can co-exist at relatively high abundances in enrichment communities with *P. tricornutum* [18, 27], indicating that even the most competitive interactions do not lead to exclusion in these cases. If *Alcanivorax* has a low growth efficiency in a community, or excretes new compounds subsequently made available to other bacteria, this could explain their co-existence.

Our results suggest the magnitude of the effect our chosen facilitative strain *Devosia* had on secondary strain *Marinobacter* is context-dependent, because the net effect was strongly positive (i.e. facilitation) in the sequential media experiment and insignificant in the microplate based on abundance differences, and slightly negative based on net carbon assimilation (all relative to no interaction controls). The experiments could have had distinct diatom-derived exometabolites pools available to the bacteria since the building material of this microplate is known to be selective in molecular diffusion dependent on molecular size [33], adsorption, and structure [34], and *P. tricornutum* exudate composition is dynamic throughout its growth [18]. For example, *Marinobacter* can consume diatom volatile organic compounds [61], which may have been relatively more abundant in the microplate due to the presence of live diatoms, potentially masking the facilitation effect of the primary strain. Our data did not support cross-feeding as the mechanism for *Devosia*’s beneficial influence, despite the prevalence of metabolic cross-feeding in bacterial interactions [47, 62–64]. Although it is possible that *Devosia* was producing a compound that we could not detect, an alternative facilitative mechanism involves degradation of inhibitory compounds. Existing literature suggests an ability of the *Devosia* genus to transform toxins or their intermediates such as deoxynivalenol [65], polycyclic aromatic hydrocarbons [66], or potentially hexachlorocyclohexane [67, 68], but these toxins could have had limited diffusivity in the microplates, leading to reduced facilitative effects. The modeling-based MRO predictions and the metabolomics-based ECI predictions had a weak correlation, as might be expected since MRO relies on gene annotation and growth on sole carbon source substrates, whereas ECI empirically measures and compares consumption of metabolites detected by LC–MS/MS. Thus, in cases where the link between resource consumption and the gene-informed metabolic pathway is not defined, we would expect a low correlation. The observed disconnect for *Devosia* (Fig. 2C) could reflect its ability to modify inhibitory metabolites through uncharacterized or non-central-metabolism gene pathways, noting that consumption includes metabolite modification. More generally, we also did not find a correlation between metabolite consumption and phylogeny of the strains assayed. This is most likely because our set of isolates are not evenly distributed across the diversity of algal-associated bacteria, but this points to the importance of using whole genomes and metabolomics methods to predict resource interactions between bacteria which can then be linked to single cell carbon incorporation.

This work demonstrates how the proximity to the host influences bacterial physiology. Using the spatially designed microplate and the algal-associated bacterium *Marinobacter*, we unexpectedly discovered that the bacteria accumulated more leucine when distant from the host as evidenced by higher single cell ^15^N enrichment. Although leucine incorporation has been a measure for estimating protein synthesis [51, 69, 70], it has also been shown recently to accumulate inside a starving bacterial cell [52], likely because of its high value due to the high metabolic cost of producing high-energy phosphate bonds [71]. Our co-culture exemplifies how two lifestyles by a single species can exist, one of which is represented as incorporating algal carbon with a high growth rate and larger cell size (*Marinobacter* in the proximal microplate well) and another as metabolically starved with smaller cells (*Marinobacter* in the distal well). This finding contributes to our previous understanding of these bacteria existing in the phycosphere microenvironment near an algal cell where diffusing exudates are the primary source of carbon in an otherwise nutrient-scarce space [72].

Our results demonstrate that facilitative interactions are important component alongside competitive microbial interactions in the phycosphere. By experimentally structuring communities spatially and temporally, we were able to decompose the complex mixture of modes of interaction and their directionality, and quantify their effects on algal carbon assimilation. Our results provide a direct link from the competitive and facilitative interactions that promote bacterial species co-existence to the modulation of algal carbon fluxes that mediate those interactions. Specifically, we found that an interaction that was facilitative in the sequential media promoted dramatic increases in algal exudate assimilation which would lead to alterations to dissolved organic matter composition. Because these compositional changes did not feedback on algal production, we propose that facilitative interactions may alter, relative to a purely competitive system [54] both the age-lability distribution of dissolved organic matter [73] and the metabolic balance of photosynthesis and respiration in the marine environment [74, 75], two fundamental controls on marine biogeochemical processes.

## Supplementary Material

SI_KimBrisson2025_ISMEJ_wraf096

KimBrisson_ISMEJ2025_SupplementaryTables_wraf096

## Data Availability

LC–MS/MS, NanoSIMS and flow cytometry data including custom scripts for visualization are available at GitHub repository (https://github.com/vbrisson/phycosphere_bacteria-bacteria_interactions). Information on the GNPS analysis and results are available at https://gnps.ucsd.edu/ProteoSAFe/status.jsp?task=94d9cced1af245bcbf6216e66fc91720 for positive mode data and https://gnps.ucsd.edu/ProteoSAFe/status.jsp?task=7a90fe1862d945ec84f8d48647f00fdf for negative mode data. Codes for draft genome scale metabolic models are provided at GitHub repository PheArrMe (https://github.com/jrcasey/PheArrMe). MALDI mass spectrometry imaging data available at https://metaspace2020.eu/project/algae-bacteria_MSI. All other data are provided in Supplementary Materials.
