# Supplementary material for: Spatially structured bacterial interactions alter algal carbon flow to bacteria
Source: ISME J. 2025 May 17;19(1):wraf096. doi: 10.1093/ismejo/wraf096 (PMC12201945; doi:10.1093/ismejo/wraf096)
Supplement: SI_KimBrisson2025_ISMEJ_wraf096 [file si_kimbrisson2025_ismej_wraf096.pdf]

Supporting Information for

Spatially structured bacterial interactions alter algal carbon flow to bacteria

Hyungseok Kim[1]*, Vanessa L. Brisson[2]*, John R. Casey[2], Courtney Swink[2], Kristina A. Rolison[2], Nathan McCall[2], Amber N. Golini[3], Trent R. Northen[3,4], Dušan Veličković[5], Peter K. Weber[2], Cullen R. Buie[1,6], Xavier Mayali[2], Rhona K. Stuart[2†]

[1] Department of Mechanical Engineering, Massachusetts Institute of Technology, Cambridge, MA, USA; [2] Physical and Life Sciences Directorate, Lawrence Livermore National Laboratory, Livermore, CA, USA; [3] Environmental Genomics and Systems Biology Division, Lawrence Berkeley National Laboratory, Berkeley, CA, USA; [4] The DOE Joint Genome Institute, Lawrence Berkeley National Laboratory, Berkeley, CA, USA; [5] The Environmental Molecular Sciences Laboratory, Pacific Northwest National Laboratory, Richland, WA, USA; [6] Department of Biological Engineering, Massachusetts Institute of Technology, Cambridge, MA, USA.

* Equal contributions.

† To whom correspondence may be addressed.

Rhona K. Stuart

Email: stuart25@llnl.gov

This PDF file includes:

Supporting text

Figures S1 to S10

SI References

Other supporting materials for this manuscript include the following:

Tables S1 to S10 (separate file)

Supporting Information Text

Supplementary Note S1. Preparation, design of porous microplate and estimation of algal exometabolites diffusion.

Device preparation. Porous microplates made of nanoporous copolymer poly(2-hydroxethyl methacrylate-co-ethylene glycol dimethacrylate) were fabricated as previously described (Ge et al 2016, Kim et al 2022, Vaiana et al 2022). Briefly, acrylic mold parts were laser cut from 1/8- and 1/4-inched acrylic sheet (Universal Laser Systems) and were attached by using acrylic (Weld-On) and epoxy (3M) adhesives. Using the acrylic mold, a polydimethylsiloxane (PDMS) mold was casted onto the acrylic and cured at 70 ºC for overnight. A prepolymer solution was prepared by mixing reagents as listed in Supplementary Table S8. The prepolymer was cast onto the PDMS mold, followed by placing a clean glass slide (75 × 50 mm2, VWR), and was polymerized by exposing to ultraviolet light with 365 nm wavelength for 15 min. Polymerized devices were detached from PDMS mold and stored in a glass jar containing 250 ml methanol. The methanol was replaced every day two times to remove remaining prepolymers. The copolymer devices were transferred to an autoclaved glass jar containing f/2-Si added with ^{13}C sodium bicarbonate and ^{15}N leucine to final concentrations of 2 mM and 10 nM, respectively, and were stored for 2-3 weeks before use.

Previous work employing the co-culture porous microplate have demonstrated that its building material, i.e., porous copolymer, enables microbial cells to communicate by exchanging their metabolites for a period of growth (~ weeks), while preventing a direct contact between adjacent cultures. We further characterized the copolymer by measuring its biomolecular diffusivity [1], tortuosity, porosity and by visualizing its structure[2]. Based on its characterization a numerical model was established estimating a concentration of algal exudates which diffused to surrounding wells during incubation[3]. In specific, concentration of dissolved organic carbon (DOC) exuded by alga *P. tricornutum* was estimated based on its growth experimentally measured in the microplate, and Fick's law of diffusion was used to calculate the DOC concentration in adjacent or surrounding wells.

In this work, the model is parametrized by changing 1) the number of surrounding wells for each distance level from the center and 2) the size ratio between center and surrounding well (Supplementary Figure S4a). The objective of this parameter study was to find an optimal configuration of a porous microplate for testing our hypothesis on sequential, bacterial uptake of algal exometabolites. Therefore, by designing the microplate we sought to maximize the amount of exometabolites reaching the bacteria, and at the same time, retain sufficient replicates for reliability. Numerical results show the concentration increased by decreasing number of surrounding wells and increasing well size ratio. The results also show that the

change in the surrounding well number had less impact than the size ratio in the concentration. These findings allowed us to configure the microplate design with six surrounding wells and the highest size ratio (Supplementary Figure S4b). This resulted in a hexagonal array of wells with their center-to-surrounding ratio ~3:1 and algal carbon flux to bacteria comparable to an algal-bacterial co-culture.

Supplementary Note S2. NanoSIMS operation conditions and derivation of total algal carbon mass incorporation in porous microplate.

NanoSIMS operation conditions. Filtered, area-cut bacterial samples were gold coated and mounted as previously described [4, 5]. Briefly, a ~60 pA primary Cs^+ beam was used to sputter a 33 x 33 µm raster for 2 cycles (131.1 s) prior to collecting 25 cycles over a 30 x 30 µm acquisition area with ~2 pA Cs^+ (~150 mm diameter). The secondary ion mass spectrometer was tuned to ~9500 mass resolving power (MRP, with aperture slit 2 and entrance slit 4), and automatic secondary ion beam centering was performed at each new location based on $^{12}C^{14}N^-$.

Derivation of the total algal carbon mass incorporated by each bacterial isolate in a porous microplate. Denoted as C_{total}, the carbon mass incorporation by either the primary strain (i) and the secondary strain (r), is defined as, for example,

$$C_i(i,r) = C_{net,\,i}(i,r) \times N_i(i,r) \times V \times M_i,$$

where $C_i(i,r)$ is the incorporation by primary strain i with the presence of secondary r, $C_{net,\,i}(i, r)$ the primary strain's average single cell C_{net}, $N_i(i, r)$ the number of the cells per volume, V the culture volume, M_i the carbon mass of a single cell (Mayali et al., Nat Comms, 2023). The relation allows to combine single-cell algal carbon uptake and bacterial abundance in the microplate co-culture. Numerical values for calculating C_{total} for each bacterial pair are detailed in Data, Materials, and Software Availability of main text.

Supplementary Note S3. Determination of minimal bacterial growth supplements for metabolic model reconstruction. Carbon sources as growth supplements were determined by using the 96-well formatted Phenotype MicroArrays™ (Biolog Inc., Hayward, CA). In brief, each of 10 bacterial isolates was inoculated to a microplate containing 190 carbon substrates in each well following manufacturer's protocol (95 substrate and one blank control per microplate). All wells contained 3% sodium chloride dissolved in deionized water. Bacterial growth was measured using plate reader for 96 hours. Absorbance time-series profiles were variable across isolates in both shape and range, and the distribution of

absorbance ranges (both linear and log transformed) was not bimodal, making a filtering threshold arbitrary. In the absence of a more sophisticated approach to filtering, a conservative classifier was implemented, wherein growth was assessed if the time-series profile met the following criteria: (a) $t_{a_i}^{max} > t_{a_i}^{min}$ and $\max a_i(t) - \min a_i(t) > f(\max a_{neg}(t) - \min a_{neg}(t))$$t_{max(ai)} > t_{min(ai)}$ and $max(a_i(t)) – min(a_i(t)) > f(max(a_{neg}(t)) – min(a_{neg}(t)))$, where $a_i(t)$ is the absolute absorbance value at time t for the ith carbon source, $a_{neg}(t)$ is the absolute absorbance at time t for the negative control, and f is a scaling factor. A visual inspection of the data was used to identify an appropriate scaling factor (f).

Supplementary Note S4. MALDI mass spectrometry imaging (MSI) and analysis. Areas with interaction colonies were excised from agar, placed onto a double-sided adhesive copper tape (3-6-1182; 3M) adhered to indium tin oxide (ITO)-coated glass slides (Bruker Daltonics, Billerica, MA), and dried at room temperature overnight prior to MALDI matrix application and MALDI MSI analysis. MALDI matrix (DHB: 40 mg/mL in 70% MeOH) was applied to the ITO-coated glass slide using an HTX TM-Sprayer (HTX Technologies). Imaging was performed on a 15 tesla (T) MALDI-Fourier transform ion cyclotron resonance-mass spectrometer (FTICR-MS; SolariX, Bruker Daltonics) equipped with SmartBeam II laser source (355 nm, 2 kHz). Spraying, imaging, and ion collection conditions are detailed in Supplementary Table S10. MS imaging data ware acquired using FlexImaging (v4.1, Bruker Daltonics), images were processed and visualized via SCiLS Lab (v2024a Premium 3D, Bruker Daltonics) from the profile datasets. The centroided dataset was automatically annotated within METASPACE (parameters given in Supplementary Table S10) and its list of features was imported back to the SCiLS after Root Mean Square (RMS) normalization. Colony borders were manually drawn based on flatbed scanner image taken prior to the MALDI matrix application. Features for each colony were filtered based on the ion signal intensity being greater than 10,000 above 3 times the intensity of blank agar.

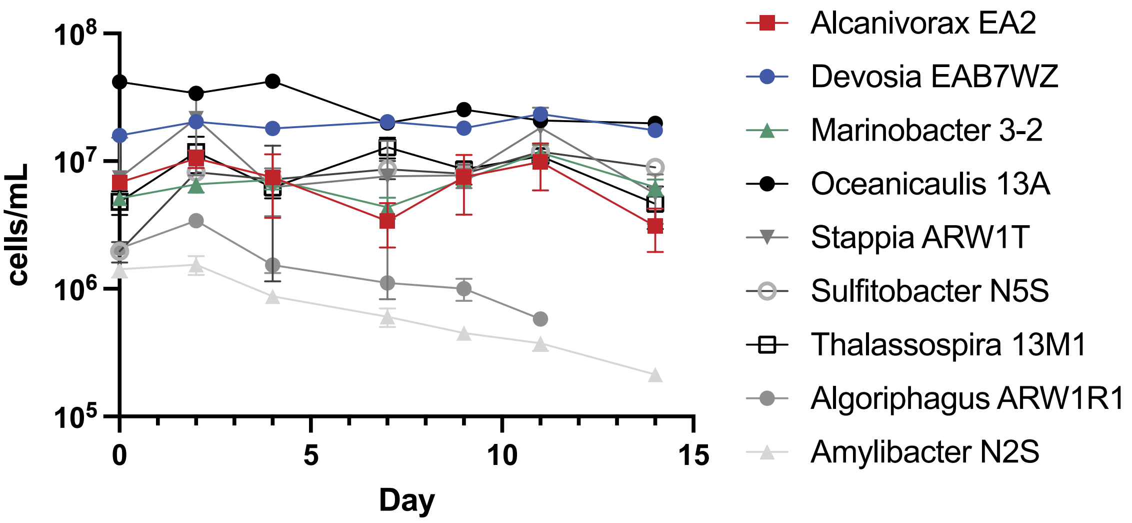

Supplementary Figure S1. Bacterial abundances for each isolate over a 14 day incubation on *P. tricornutum* spent media. Points represent average abundance from flow cytometric counts from biological triplicates. Error bars represent one standard deviation. Incubations of each isolate was conducted in 4 mL volumes in glass tubes, with removal of 100 µL every other day for flow cytometric counts. Media preparation, culturing, and growth conditions identical to those described in the "*Untargeted metabolomics of exometabolite production and consumption*" Methods section.

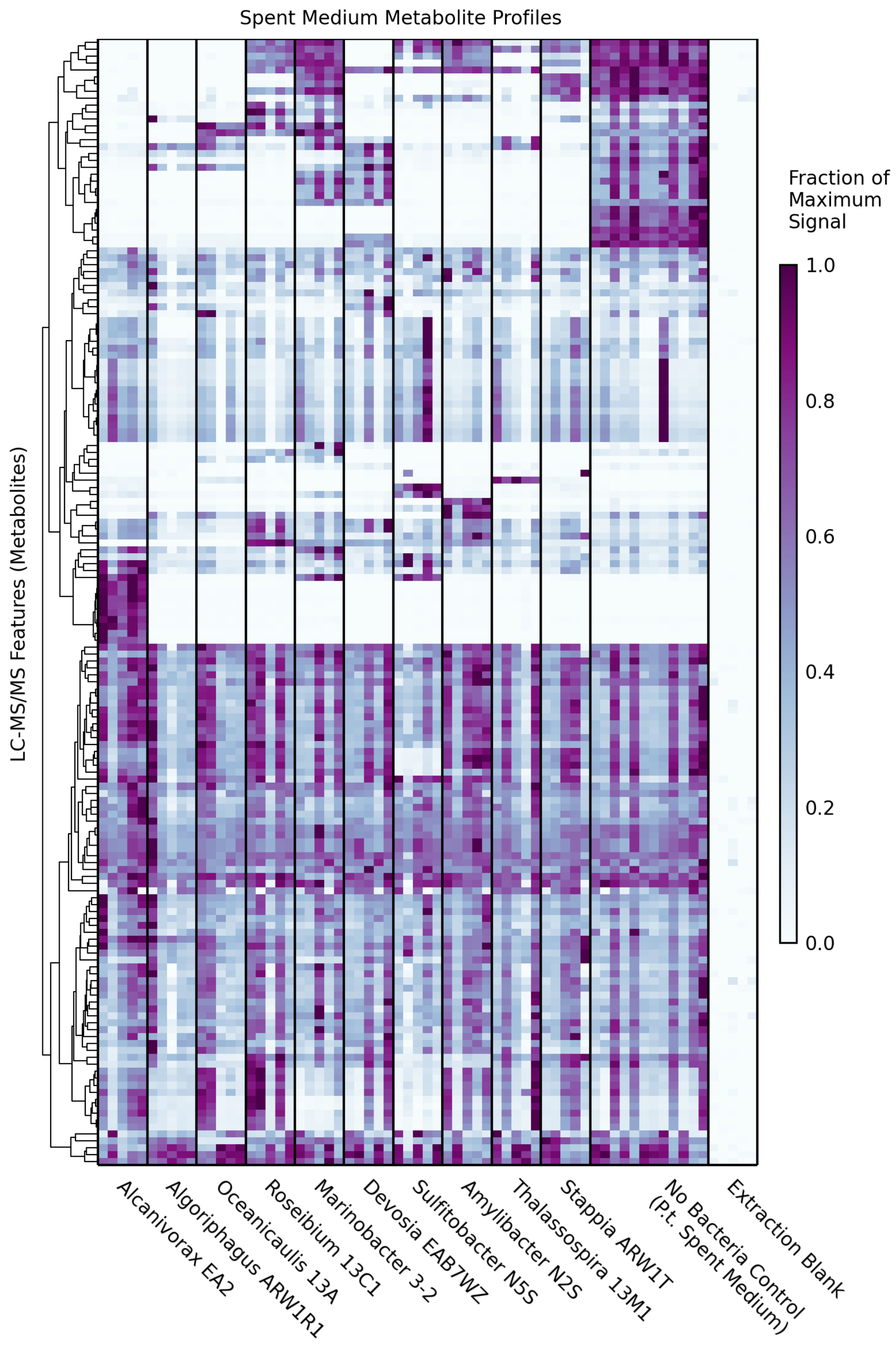

Supplementary Figure S2. LC-MS/MS metabolomics profiles for bacterial isolated grown on spent medium from *P. tricornutum*. Each row represents one LC-MS/MS feature (*m/z* and retention time), and each column represents one sample. Samples are grouped by the bacterial isolate, with all 5 replicate samples shown. Rows are grouped based on hierarchical clustering, as indicated in the dendrogram on the left.

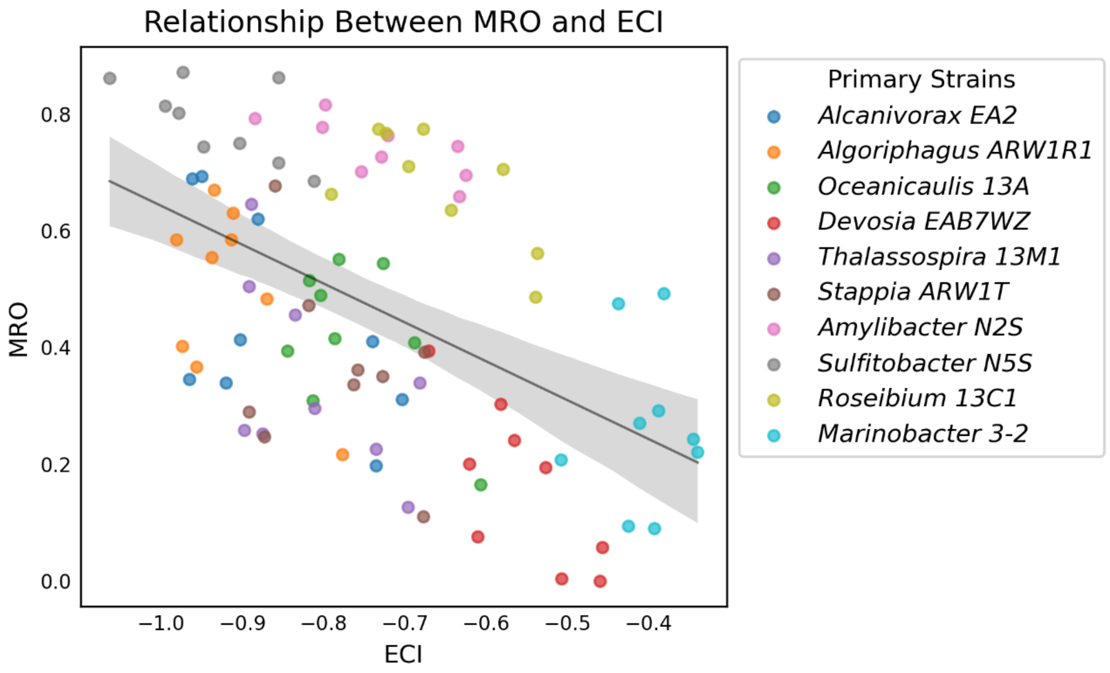

Supplementary Figure S3. Relationship between metabolic resource overlap (MRO) and estimated competitive interactions (ECI). Each point represents one primary-secondary strain pair, and the color is based on the primary strain. The diagonal line shows the Pearson correlation ($R^2 = 0.25$, $P = 6 \times 10^{-7}$), and the grey area indicates the 95% confidence interval.

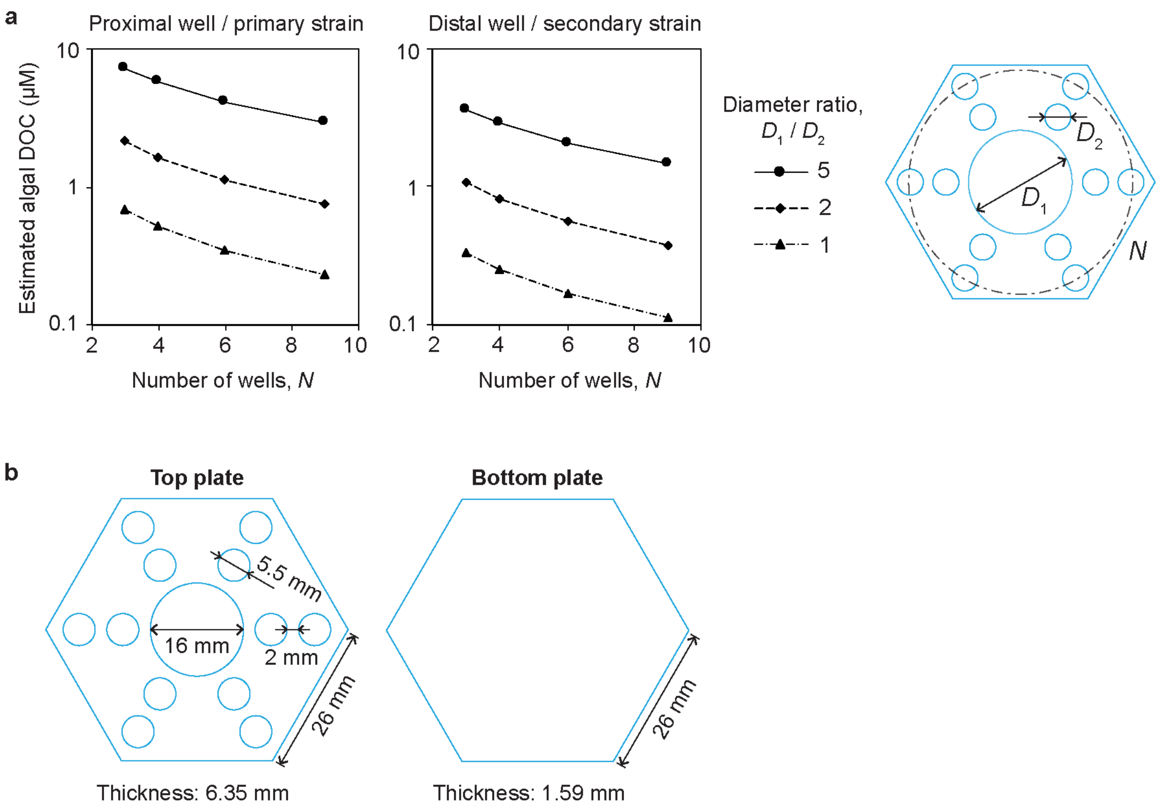

Supplementary Figure S4. Design of porous microplate for algal-bacterial co-culture. (a) Estimation of log-scaled algal dissolved organic carbon (DOC) concentration diffused to surrounding wells, parametrized by a ratio between center and surrounding wells and the number of surrounding wells for each distance level (proximal, left; distal, right). Concentrations are calculated based on abundance of *P. tricornutum* incubated in the porous microplate for twenty days (see Supplementary Note S1 for details). (b) Final dimension of porous microplates used in this study.

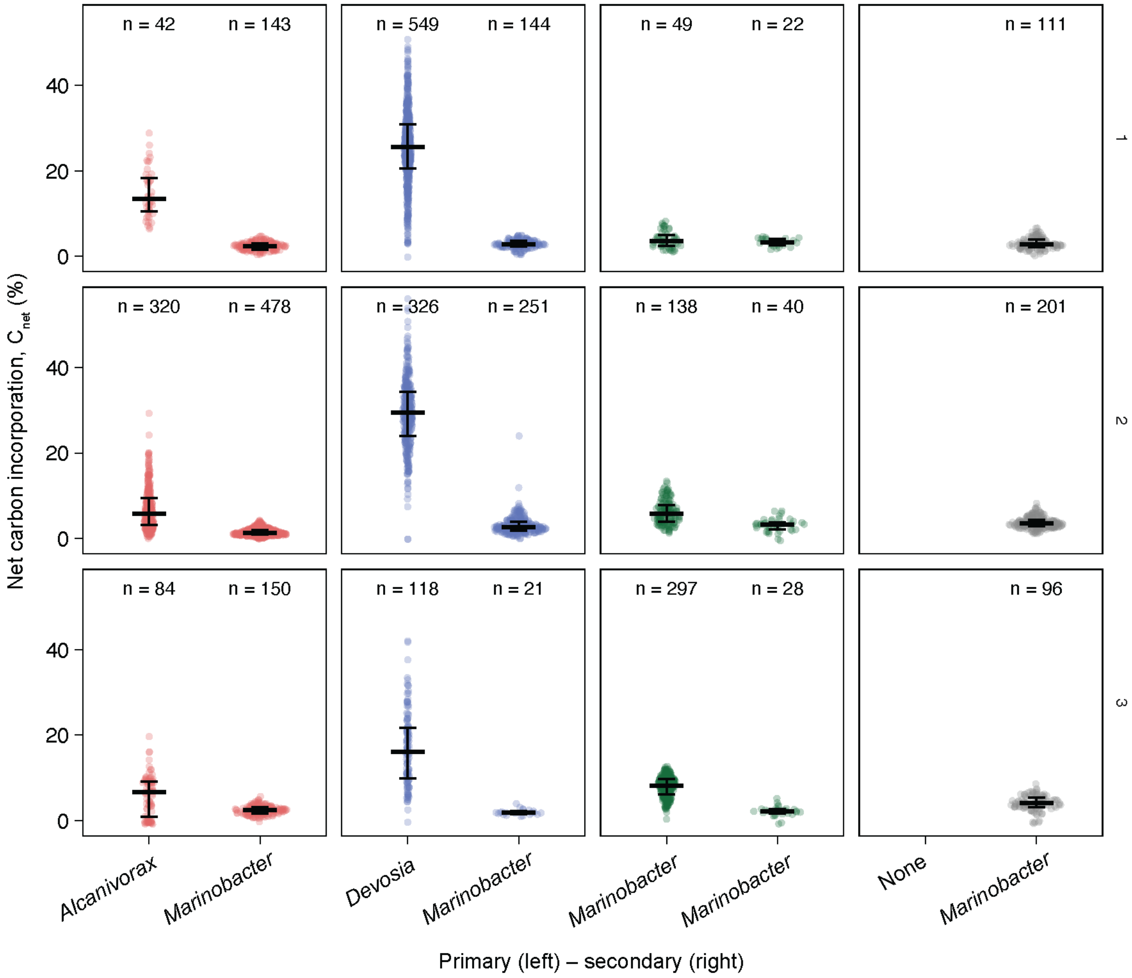

Supplementary Figure S5. Net carbon incorporation (C_{net}) of primary and secondary bacterial strains co-cultured with *P. tricornutum*, organized in columns, in three porous microplates measured using single-cell isotope tracing and NanoSIMS, organized in rows. Black middle line and error bar indicate median and interquartile ranges, respectively. Number of single cells (n) analyzed for each primary treatment, well location, and microplate is displayed.

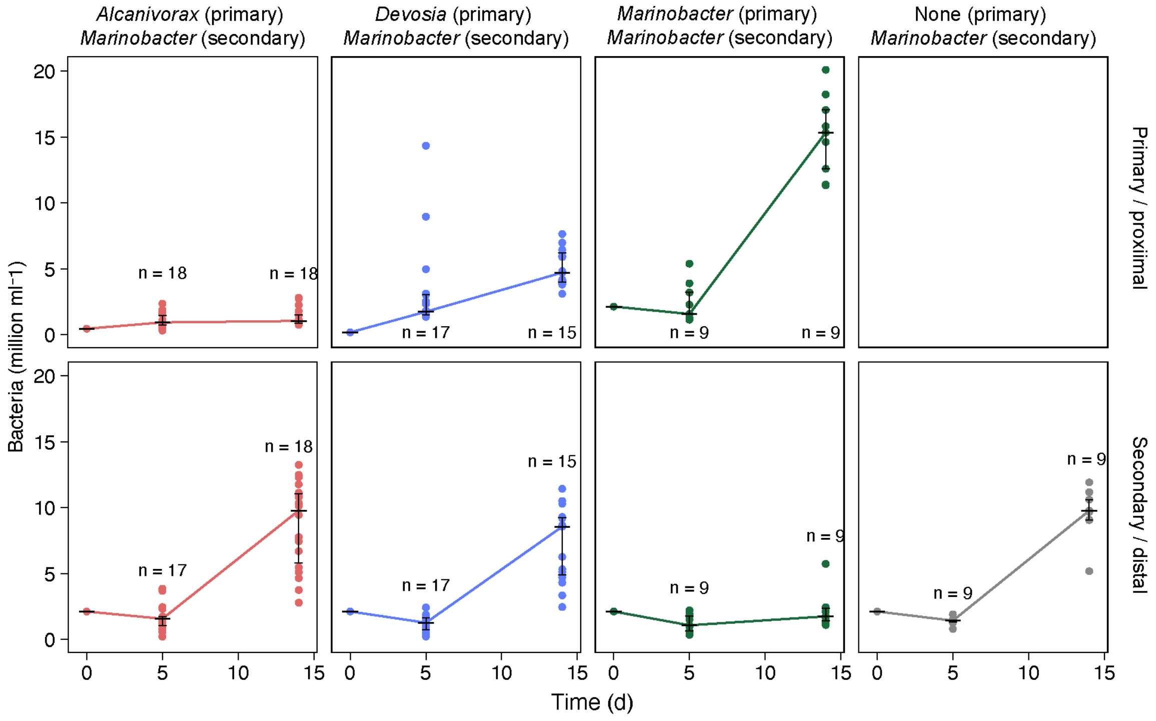

Supplementary Figure S6. Abundances of primary and secondary bacterial strains co-cultured with *P. tricornutum* in porous microplates. Each point represents measurement using flow cytometry of samples from each well of the microplate on days 0, 5, 14 of incubation. Black lines indicate median (thick) and interquartile ranges (error bar). Number of samples (n) analyzed for each treatment and well location is displayed.

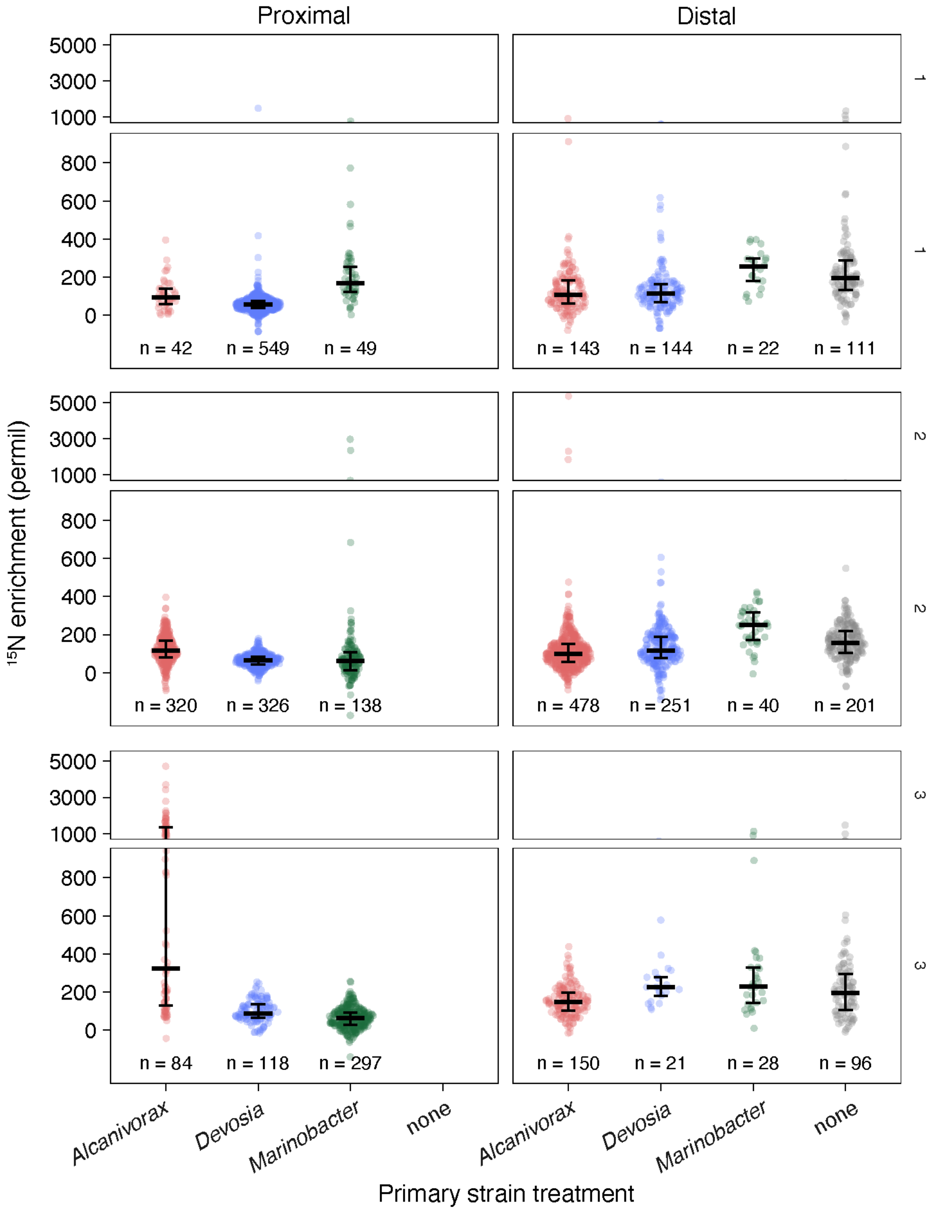

Supplementary Figure S7. Enrichment of ^{15}N by bacteria co-cultured with *P. tricornutum* in three porous microplates. The enrichment was measured using single-cell isotope tracing and NanoSIMS. Black middle line and error bar indicate median and interquartile ranges, respectively. Number of single cells (n) analyzed for each treatment, location and microplate is displayed.

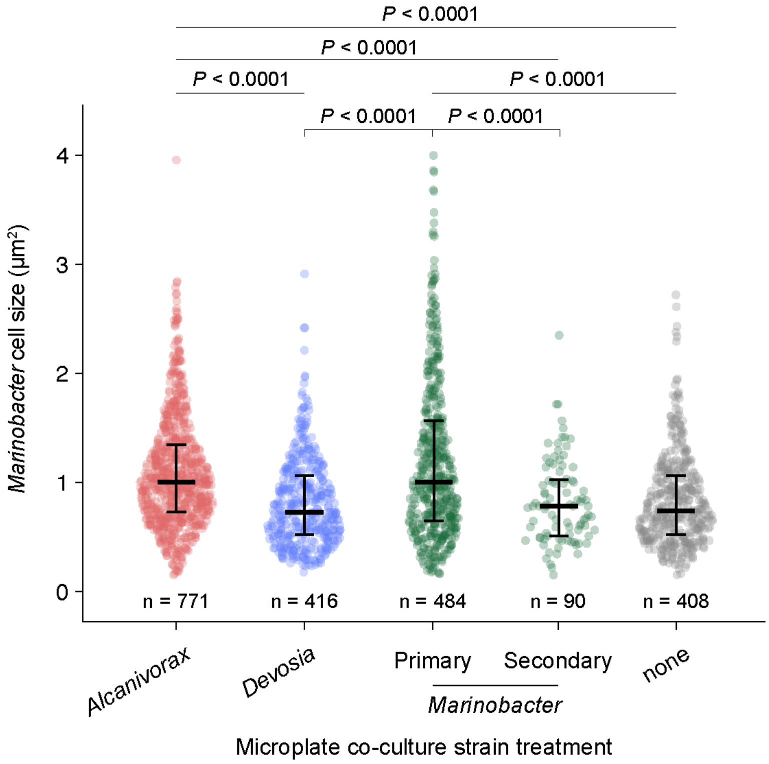

Supplementary Figure S8. Individual cell size of primary/secondary strains co-cultured with *P. tricornutum* in porous microplate. Black lines indicate median (thick) and interquartile ranges (error bar). Each point represents measurement of single cell area region of interest (ROI) from nanoSIMS images.

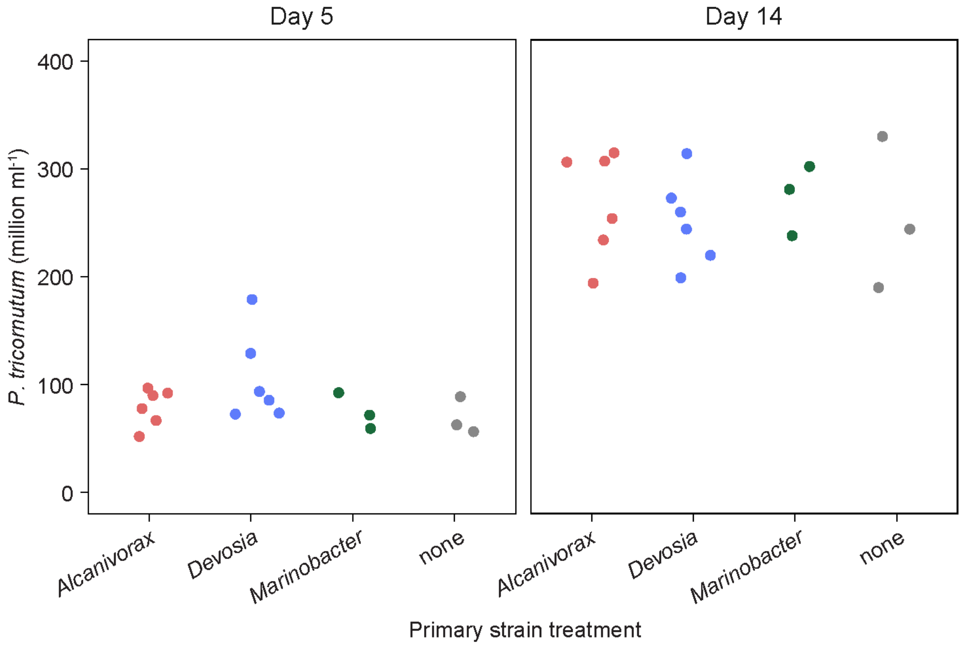

Supplementary Figure S9. Abundance of *P. tricornutum* incubated in porous microplate. *P. tricornutum* was incubated for day 5 and 14 and co-cultured with different bacterial primary strain and secondary strain, located in proximal and distal wells respectively.

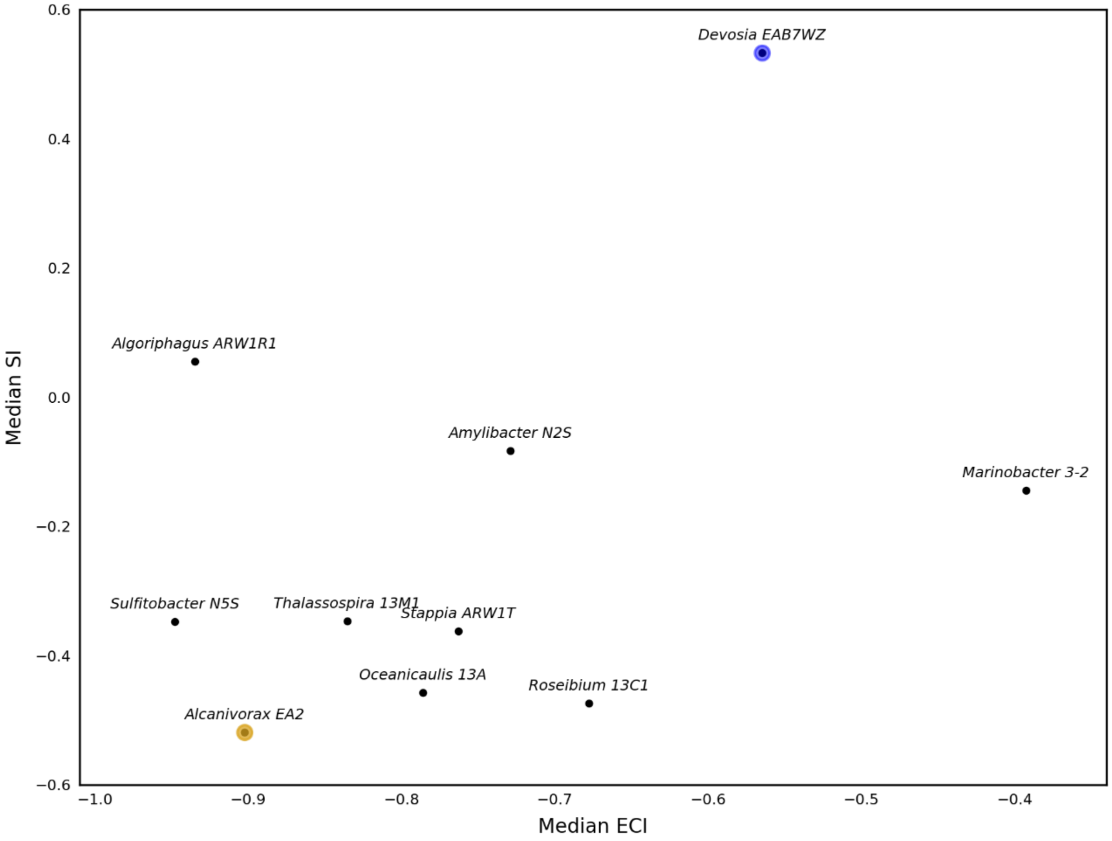

Supplementary Figure S10. Median SI and ECI for primary strains. Primary strains studied in the porous microplate experiment are highlighted in yellow and blue for *Alcanivorax* and *Devosia* respectively.

Supplementary Table S1 (separate file). LC-MS/MS feature peak heights.

Supplementary Table S2 (separate file). Putative identifications for metabolite features based on spectral matches in GNPS.

Supplementary Table S3 (separate file). Flow cytometry counts from the spent medium exchange experiment used to calculate the sequential interaction strength (SI).

Supplementary Table S4 (separate file). Top five matches from sequence alignments between *Marinobacter* genome and bacterial leucine transporter gene.

Supplementary Table S5 (separate file). Feature detection with matrix assisted laser desorption/ionization (MALDI) imaging.

Supplementary Table S6 (separate file). Instrument information and LC-MS/MS parameters for metabolomic analysis.

Supplementary Table S7 (separate file). MZMine analysis parameters for feature identification.

Supplementary Table S8 (separate file). Reagents and their composition for copolymer poly(2-hydroxethyl methacrylate-co-ethylene glycol dimethacrylate) (HEMA-EDMA).

Supplementary Table S9 (separate file). Parameters for operating flow cytometry and software.

Supplementary Table S10 (separate file). Parameters and conditions for operating MALDI mass spectrometry imaging (MSI) and analysis.

SI References

1. Ge Z, Girguis PR, Buie CR. Nanoporous microscale microbial incubators. *Lab Chip*. 2016;**16**:480-88
2. Vaiana CA, Kim H, Cottet J *et al.* Characterizing chemical signaling between engineered “microbial sentinels” in porous microplates. *Mol Sys Biol*. 2022;**18**:e10785 https://doi.org/https://doi.org/10.15252/msb.202110785
3. Kim H, Kimbrel JA, Vaiana CA *et al.* Bacterial response to spatial gradients of algal-derived nutrients in a porous microplate. *ISME J*. 2022;**16**:1036-45 https://doi.org/10.1038/s41396-021-01147-x
4. Mayali X, Samo TJ, Kimbrel JA *et al.* Single-cell isotope tracing reveals functional guilds of bacteria associated with the diatom *Phaeodactylum tricornutum*. *Nat Commun*. 2023;**14**:5642 https://doi.org/10.1038/s41467-023-41179-9
5. Samo TJ, Kimbrel JA, Nilson DJ *et al.* Attachment between heterotrophic bacteria and microalgae influences symbiotic microscale interactions. *Environl Microbiol*. 2018;**20**:4385-400
